# Rapid and accurate detection of *KRAS* mutations in colorectal cancers using the isothermal-based optical sensor for companion diagnostics

**DOI:** 10.18632/oncotarget.20038

**Published:** 2017-08-08

**Authors:** Choong Eun Jin, Seung-Seop Yeom, Bonhan Koo, Tae Yoon Lee, Jeong Hoon Lee, Yong Shin, Seok-Byung Lim

**Affiliations:** ^1^ Department of Convergence Medicine, Asan Medical Center, University of Ulsan College of Medicine, Biomedical Engineering Research Center, Asan Institute of Life Sciences, Asan Medical Center, Seoul, Republic of Korea; ^2^ Division of Colon and Rectal Surgery, Asan Medical Center, University of Ulsan College of Medicine, Seoul, Republic of Korea; ^3^ Department of Technology Education, Chungnam National University, Daejeon, Republic of Korea; ^4^ Department of Electrical Engineering, Kwangwoon University, Seoul, Republic of Korea

**Keywords:** KRAS mutations, colorectal cancer, companion diagnostics, silicon bio-photonic sensor, rapid isothermal DNA amplification

## Abstract

Although *KRAS* mutational status testing is becoming a companion diagnostic tool for managing patients with colorectal cancer (CRC), there are still several difficulties when analyzing *KRAS* mutations using the existing assays, particularly with regard to low sensitivity, its time-consuming, and the need for large instruments. We developed a rapid, sensitive, and specific mutation detection assay based on the bio-photonic sensor termed ISAD (isothermal solid-phase amplification/detection), and used it to analyze *KRAS* gene mutations in human clinical samples. To validate the ISAD-KRAS assay for use in clinical diagnostics, we examined for hotspot *KRAS* mutations (codon 12 and codon 13) in 70 CRC specimens using PCR and direct sequencing methods. In a serial dilution study, ISAD-KRAS could detect mutations in a sample containing only 1% of the mutant allele in a mixture of wild-type DNA, whereas both PCR and direct sequencing methods could detect mutations in a sample containing approximately 30% of mutant cells. The results of the ISAD-KRAS assay from 70 clinical samples matched those from PCR and direct sequencing, except in 5 cases, wherein ISAD-KRAS could detect mutations that were not detected by PCR and direct sequencing. We also found that the sensitivity and specificity of ISAD-KRAS were 100% within 30 min. The ISAD-KRAS assay provides a rapid, highly sensitive, and label-free method for *KRAS* mutation testing, and can serve as a robust and near patient testing approach for the rapid detection of patients most likely to respond to anti-EGFR drugs.

## INTRODUCTION

Colorectal cancer (CRC) is the third most common cancer among men (746,000 cases, 10.0% of the total) and the second most common cancer among women (614,000 cases, 9.2% of the total) [1–5]. More than 1 million patients are diagnosed annually, of which 50% will develop metastatic disease [1, 2, 6, 7]. CRCs can be classified based on the presence of mutations in oncogenes such as *KRAS*, *BRAF,* and *PIK3CA*. Among the affected oncogenes, *KRAS* mutations are present in 35–40% of CRC cases, and the presence of a *KRAS* mutation is an early phenomenon in CRC [3–5]. The *KRAS* proto-oncogene encodes a guanosine triphosphate (GTP)/guanosine diphosphate (GDP) binding protein that acts downstream of epidermal growth factor receptor (EGFR) in the RAS/RAF/MAPK pathway [3, 6]. Ninety percent of *KRAS* mutations occur in codons 12 and 13 of exon 2, and these mutations are widely accepted as predictive biomarkers for the response to treatment with anti-EGFR drugs in patients with metastatic CRC [8–11]. Cetuximab and panitumumab are monoclonal anti-EGFR drugs that bind to EGFR with high affinity and competitively inhibit ligand binding, thus leading to the inhibition of phosphorylation and the subsequent activation of downstream signaling pathways in patients with *KRAS* wild-type tumors [3, 6, 9]. However, the presence of a *KRAS* mutation negatively affects the potential efficacy of therapies involving EGFR inhibition [12–14]. A recent study indicated that a higher-sensitivity *KRAS* mutation testing method could help identify patients who had a poor response to anti-EGFR therapy in CRC [6, 15–16]. Therefore, *KRAS* mutation plays an important role as a prognostic biomarker for CRC survival. Hence, the methodological aspects of *KRAS* testing and the types of assays are important, and a good balance between accuracy and practicality of the testing method is desired [15].

The need for additional genetic information regarding the *KRAS* mutation is becoming critical in order to make appropriate clinical decisions before or after surgery, and to design future clinical trials [3–5]. Thus far, the direct sequencing method has been used as a gold standard method for obtaining information on the *KRAS* mutation status from the patient's tumor tissues. However, the sequencing techniques are too complex, time-consuming, and expensive for routine pre-therapeutic screening programs [16–19]. Moreover, the complexity of the clinical samples leads to the overlooking of mutations by the direct sequencing methods, as the sample contains only a small subpopulation of mutant cells mixed with an excess of normal cells. For the conclusive determination of a mutant subpopulation by direct sequencing, the mutant cell needs to be present at a minimum concentration of ~30% of the total genetic content [20–21]. Hence, high sensitivity in relation to *KRAS* mutation assays is crucial in minimizing the risk of false negative results in tumor specimens containing low quantities of mutated DNA [20, 22]. Alternatively, many new methods based on polymerase chain reaction (PCR) for detecting *KRAS* mutations have been established [23–24], although these methods require fairly complex, time-consuming, and accurate thermo-cycling instrumentation [25]. Additionally, high-resolution melting (HRM) analysis is performed to detect the *KRAS* mutations, but the sensitivity of this method is known to be only 5–6% of the mutant cells [26–27]. Digital PCR is also applied to detect the *KRAS* mutations. This approach enables multiple target detection and accurate quantification, but is far from infallible because it uses expensive equipment and testing reagents, has a low-throughput format, and the technology requires further development [9, 25, 28]. The BEAMing technique and IntPlex methods display limited sensitivity, ranging from 1% to 5%, in relation to the detection of rarely mutated *KRAS* alleles; moreover, some other disadvantages limit the application of these methods in clinical oncology [29, 30] These techniques are more accurate and sensitive than sequencing, but are still time consuming, complicated, and unsuitable for routine clinical examination.

Recently, biosensors have become a promising and interesting innovative detection technique in cancer diagnostics, as they exhibit superior analytical performance and real-time measurement, as compared to the existing standard methods [31–34]. In particular, optical sensors, including optical fibers and waveguide devices, have been used to improve the detection sensitivity by increasing the interactions between the guided light and the sensor surface [35–36]. As nucleic acid (DNA or RNA)-based biosensors use a highly selective sequence from single-stranded DNA or RNA as bio-recognition elements, many optical biosensors based on surface plasmon resonance (SPR), optofluidic ring resonator (OFRR), and silicon microring resonator (SMR) for nucleic acids have been assessed to examine the interactions of the biomolecules [35–37]. SMR sensors have been well-established silicon micro-fabrication process and developed as a sensitive bio-molecule detection method without requiring additional substances for enhancement of obtaining signal [36–38]. It is a refractive index-based sensor provides highly sensitive, label-free, real-time multiplexed detection of biomolecules near the sensor surface [39–40]. The sensors transduce the presence of target molecules based on binding-induced changes in the refractive index proximal to the waveguide surface [36–38]. Recently, the SMR sensors have been used to detect several bio-molecules including protein, nucleic acids, aptamer, telomerase, by monitoring a shift in the resonant wavelength [35, 37, 41]. In addition, SMR sensors are much more compact sensing platform than other designs of optical sensors that can be potentially reduced sample volume and assay time [34]. Furthermore, the fabrication of the bio-optical sensors by complementary metal oxide-semiconductor (CMOS) technology minimizes costs [35, 42]. Using the SMR sensor, the interaction of the biomolecules leads to a change in the mass and refractive index of the sensor surface layer [35–36, 43]. Hence, these biosensors offer several advantages, such as label-free sensing, low detection time, portability, and multiplex detection. However, the clinical utility of biosensors is being assessed in studies with large clinical samples. Nevertheless, there is a high demand of reliable biosensors for the rapid detection of genetic alteration in the clinical setting, which could improve the clinician's decision and treatment strategy, and thus subsequently improve the patient's survival rate.

We have developed an isothermal solid-phase amplification/detection (ISAD) assay that can amplify and detect single nucleotide polymorphisms (SNPs) with high sensitivity, specificity, and rapidity (30 min) under isothermal cycling using a combination of recombinase polymerase amplification (RPA, which can reduce the use of instruments and reaction time) and the SMR sensor (which is a refractive index-based optical sensor that enables highly sensitive, label-free, real-time multiplexed detection) [44]. The advantage of RPA is that it can form a complex consisting of a primer and a recombinase enzyme to extend the DNA, which negates the need for a polymerase and cycle repetition [39, 45]. Due to these advantages of RPA, it has been applied for the nucleic acid amplification by integration with many emerging techniques such as microfluidic, lateral flow paper, digital SlipChip and so on [46–49]. Thus, the isothermal RPA technique indicates that it could be useful for the amplification of DNA molecule in human diseases.

In the present study, we developed the ISAD-KRAS assay to detect 2 *KRAS* hotspot mutations (Exon 12 & 13), and showed the sensitive and specific detection of these sequence changes in the tumor samples. Using this sensitive and rapid assay, we aimed to accurately and rapidly detect *KRAS* mutations in 70 tumor tissues from CRC patients. Using the ISAD-KRAS assay, we identified additional the *KRAS* mutations 55/70 (78.6%) compared with PCR (67.1%) and direct sequencing (61.4%). The ISAD-KRAS assay, with both G12D and G13D, showed a value of 100% and 100% for sensitivity and specificity, respectively. In contrast, the sensitivity and specificity of PCR (91.6% and 100% of G12D, 96.2% and 100% of G13D) and sequencing (87.5% and 100% of G12D, 84.6% and 100% of G13D) were lower than that of the ISAD-KRAS. This assay is fast (< 30 min), has high sensitivity and specificity, and is cost-effective, as compared to PCR and direct sequencing, for detecting *KRAS* mutation in clinical specimens. The main goal of this study was to develop a reliable assay in clinical samples for the rapid screening of *KRAS* mutations that has the potential for companion diagnostics testing in patients.

## RESULTS

### Testing the KRAS mutations with the PCR and ISAD-KRAS assays

**Figure 1:**
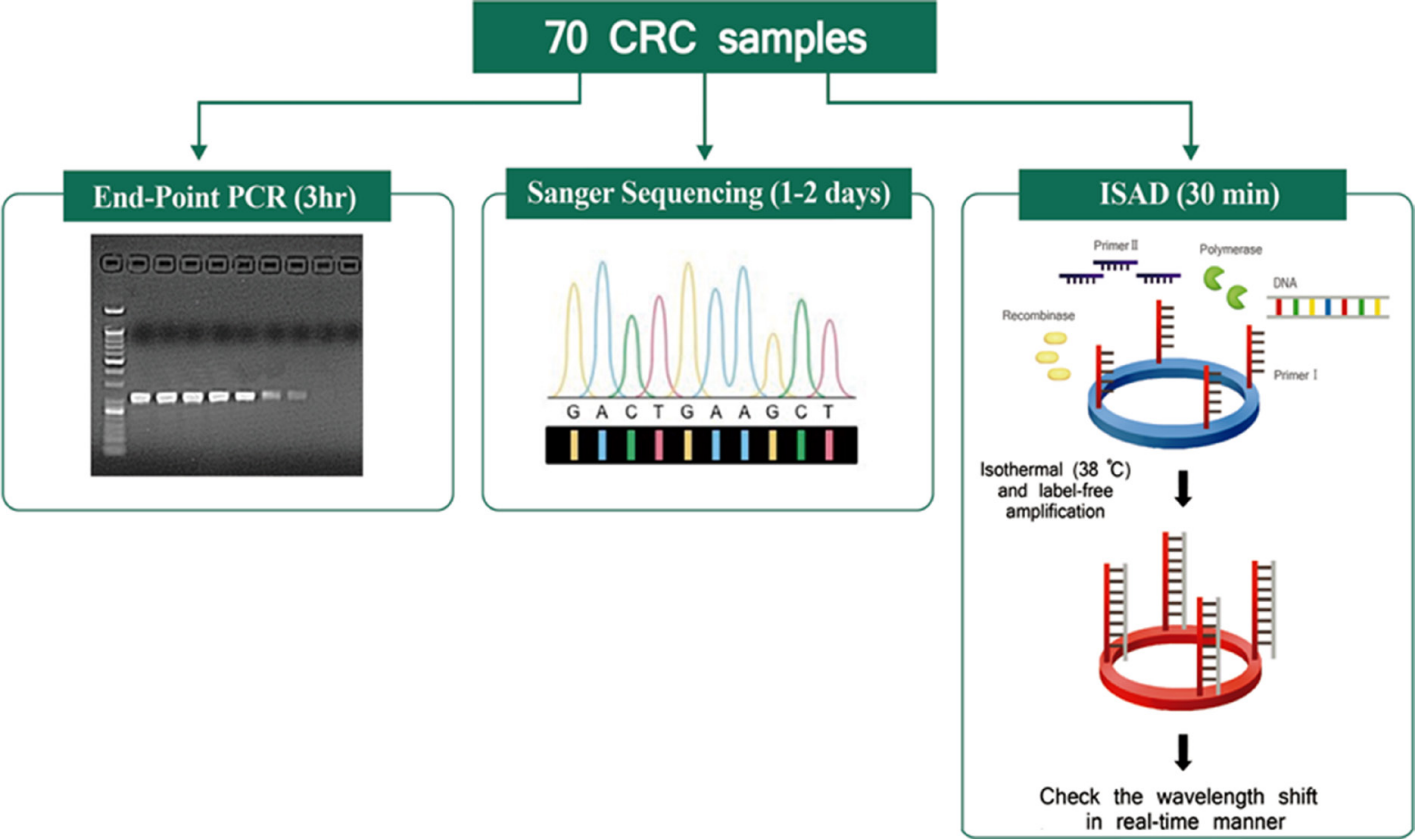
Schematic comparison of three assays A total of 70 colorectal cancer samples were tested for the *KRAS* mutations using the indicated detection assays, including end-point PCR (3 h), direct sequencing (1–2 days), and the ISAD-KRAS assay (30 min).

**Figure 2:**
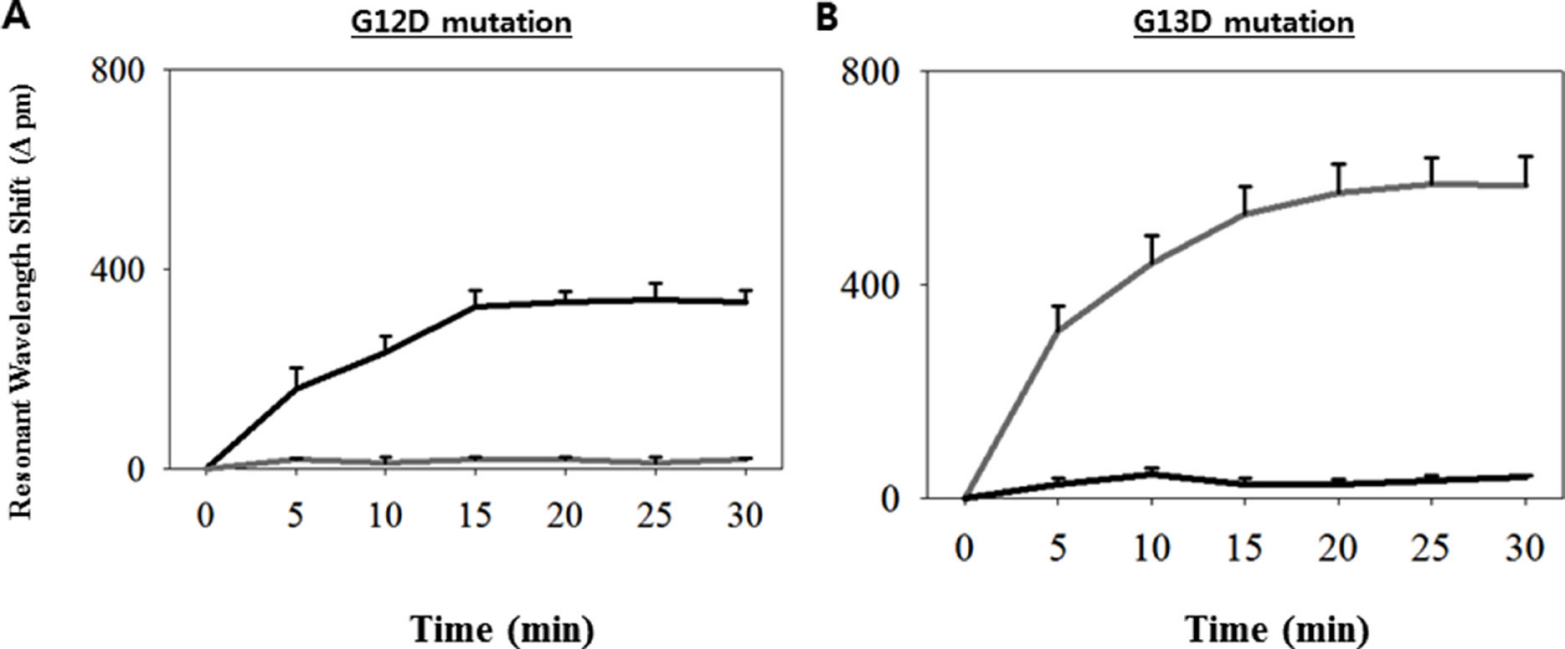
Characterization of the ISAD-KRAS assays with the *KRAS* mutant primers (**A**–**B**) Resonance wavelength shift showing the results of the amplification of the G12D mutation (left) and G13D mutation (right) with the ISAD-KRAS assay within 30 min, along with the DNAs extracted from both the AGS cells containing G12D mutation (black) and HCT116 cells containing G13D mutation (gray). The error bars indicate the standard deviation of the mean, based on at least 3 independent experiments.

In this study, we aimed to validate the clinical utility of the ISAD-KRAS assay (developed for the rapid and accurate detection of genomic variations in genes) in the decision making by clinicians regarding the appropriate treatment of patients. Accordingly, we performed the ISAD-KRAS assay, PCR, and direct sequencing to compare the clinical utility of mutation testing of the *KRAS* gene in 70 CRC samples (Figure 1). Before testing the clinical samples using our mutant primer sets to detect the G12D and G13D *KRAS* mutations, basic experiments were performed using genomic DNA obtained from 2 cancer cell lines, including AGS cells (which carry a G12D mutation but no G13D mutation) and HCT116 cells (which carry a G13D mutation but no G12D mutation). Two primer sets were used to test the samples containing the mutations of the *KRAS* gene. One of the primer sets is the wild-type primer set, which was designed by using the conventional sequence of *KRAS*, and the other is the mutant primer set, which was designed by using the mutation sequence of *KRAS* (Supplementary Table 1). When the wild-type primers were used with the PCR method, both the wild-type and mutant templates were amplified. In contrast, when the mutant primers were used, the mutant templates were amplified, and the same primer was incapable of amplifying the wild-type target (Supplementary Figure 1A–1B). It was clearly observed that the exponential amplification of a non-specific target was inhibited by the mutant primer sets. Thereafter, we used the *KRAS* mutant primers with the ISAD-KRAS assay to identify the G12D and G13D mutations of *KRAS* (Figure 2A–2B). The SMR sensors were first immobilized with either the G12D or G13D mutant primers, and both templates (G12D in AGS cells and G13D in HCT cells) were introduced for the ISAD-KRAS assay. When the G12D mutant primers were used, the wavelength shift in the G12D mutant cells from AGS was found to be approximately 335 pm, although the wavelength shift in the no G12D mutant cells from HCT116 was found to be approximately 20 pm. In contrast, when immobilizing the G13D mutant primers on SMR, the wavelength in the G13D mutant cells from HCT116 was shifted by approximately 586 pm, as compared to that in the no G13D mutant cells from AGS (approximately 40 pm). Thus, Figure 2 shows that ISAD-KRAS and mutant primers can rapidly distinguish single nucleotide differences between the target and non-target sequences within 10–30 min, thus enabling the reliable discrimination of mutants from wild-type populations.

### Detection limit of the KRAS mutation in mixed-cell populations using ISAD-KRAS

**Figure 3:**
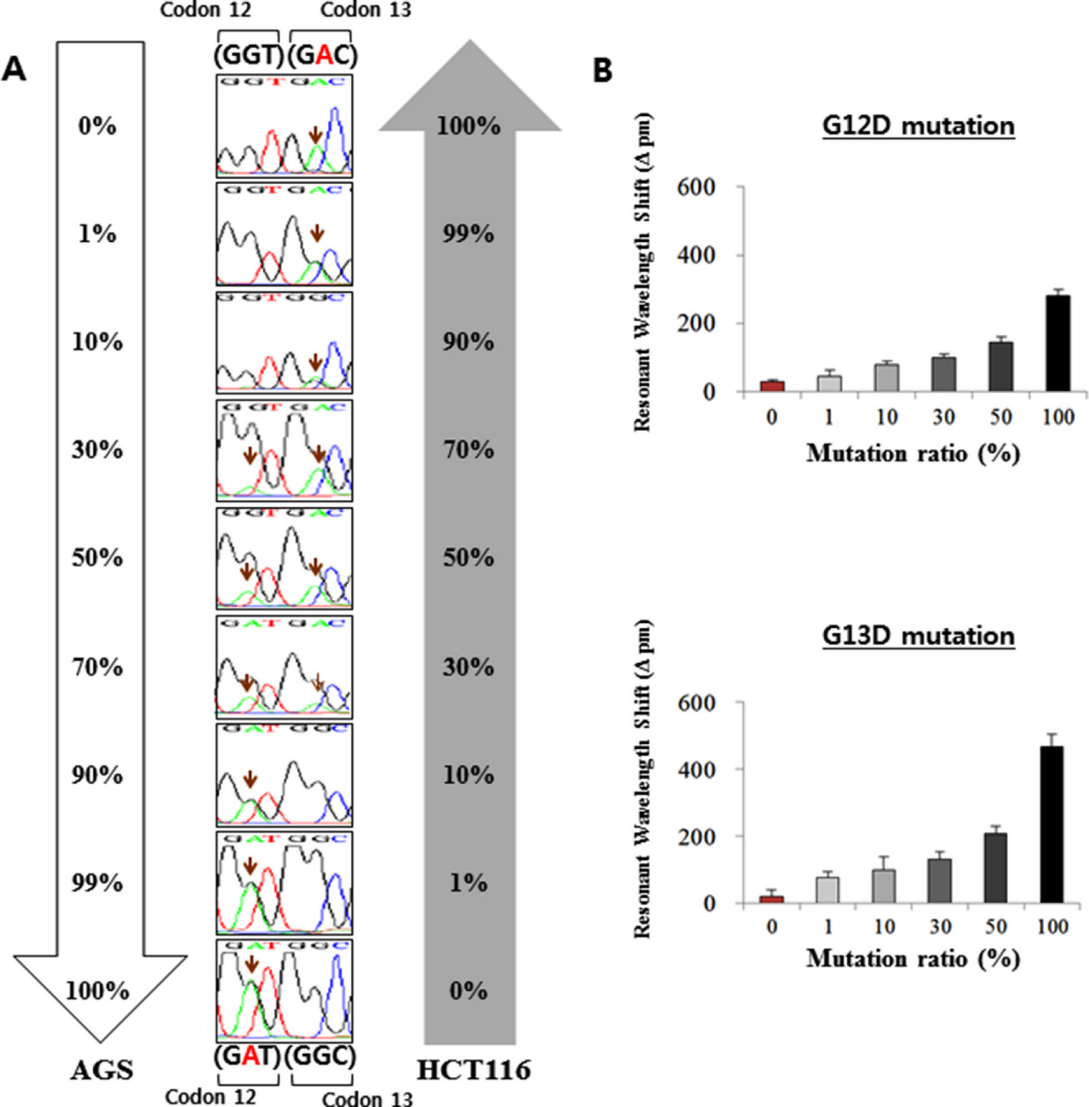
Analysis of the *KRAS* mutations with serially diluted mixed cells The AGS cells (containing the G12D mutation) were diluted with HCT116 (containing the G13D mutation) cells. The percentages of the mutant cells were 0%, 1%, 10%, 30%, 50%, and 100%. (**A**) the results of direct sequencing, following the dilution of the AGS and HCT116 cell lines. (**B**) Shift in the resonant wavelength using the ISAD-KRAS assay in the diluted mutation cells with either the G12D mutant primer (upper) or the G13D mutant primer (lower). The error bars indicate the standard deviation of the mean, based on at least 3 independent experiments.

Given that the cancer samples are heterogeneous, a useful assay for the detection of *KRAS* mutations should be able to accurately detect the mutations in mixed cells with wild-type populations. In order to test this capability of the ISAD-KRAS assay, we used serially diluted samples of mixed cells. To test the G12D mutation, AGS cells carrying the *KRAS* G12D mutation were diluted with HCT116 cells carrying no *KRAS* G12D mutation, and were used as wild-type cells. In contrast, to test the G13D mutation, HCT116 cells carrying the *KRAS* G13D mutation were diluted with AGS cells carrying no *KRAS* G13D mutation, and were used as wild-type cells. Genomic DNA was obtained from the cell mixtures containing 0% to 100% of the mutant cells in the background of wild-type cells. All genomic DNAs from the samples were examined via PCR, direct sequencing, and ISAD-KRAS (Figure 3 and Supplementary Figure 1C–1D). When PCR was used to amplify the G12D or G13D mutation, the mutant targets could be amplified within 2–3 h, particularly when the concentration of mutant cells was > 30% (Supplementary Figure 1C–1D). When direct sequencing was used to detect the G12D and G13D mutations, the mutant sequences could be identified within 1–2 days, particularly when the concentration of mutant cells was > 30% (Figure 3A). Consistent with previous reports, PCR and direct sequencing could not detect the presence of the mutant allele at a low copy number (< 30%) in wild-type populations. Figure 3B shows that the mutant sequences for G12D and G13D could be rapidly detected using ISAD-KRAS, by measuring the differences in the wavelength shifts in the samples containing 1% to 100% of the mutant cells. The ISAD-KRAS assay could detect the mutant allele in 30 min, at a concentration of only 1% mutant cells in the background of wild-type DNA (Figure 3B). Thus, the detection limit of the ISAD-KRAS assay in mixed cell samples is superior to those of the conventional methods.

### Sensitivity and specificity of ISAD-KRAS in clinical samples

To validate the clinical utility, as well as the rapidity and accuracy of the ISAD-KRAS assay using clinical samples, we randomly selected 70 frozen tissues samples from CRC patients in Bio-Resource Center (BRC) of Asan Medical Center. A total of 70 samples, including 24 samples with the G12D mutation (34.3%), 26 samples with the G13D mutation (37.1%), and 20 samples with no mutation (28.6%), were examined at the BRC and were used as reference (Supplementary Table 2). In these samples, the ability of the ISAD-KRAS assay to diagnose specific mutations within 30 min was assessed, along with both the PCR and direct sequencing methods. When the PCR method was used for the 70 clinical samples, the G12D mutation was detected in 22 samples (31.4%) and the G13D mutation was detected in 25 samples (35.7%) (Supplementary Table 2). When the direct sequencing method was used for the 70 clinical samples, the G12D mutation was detected in 21 samples (30%) and G13D mutation was detected in 22 samples (31.4%). Both methods are relatively less sensitive than the reference result from the BRC (Supplementary Table 2). Thereafter, the DNAs extracted from the 70 CRC samples were assessed via ISAD-KRAS, by immobilizing either the G12D or G13D mutant primers on the optical sensors. When the G12D mutant primers were immobilized on the sensor, a resonant wavelength shift in the samples containing only the G12D mutation of > 600 pm was considered a positive result, whereas a resonant wavelength shift in the samples containing either no mutation or G13D mutation < 600 pm was considered a negative result.

**Figure 4:**
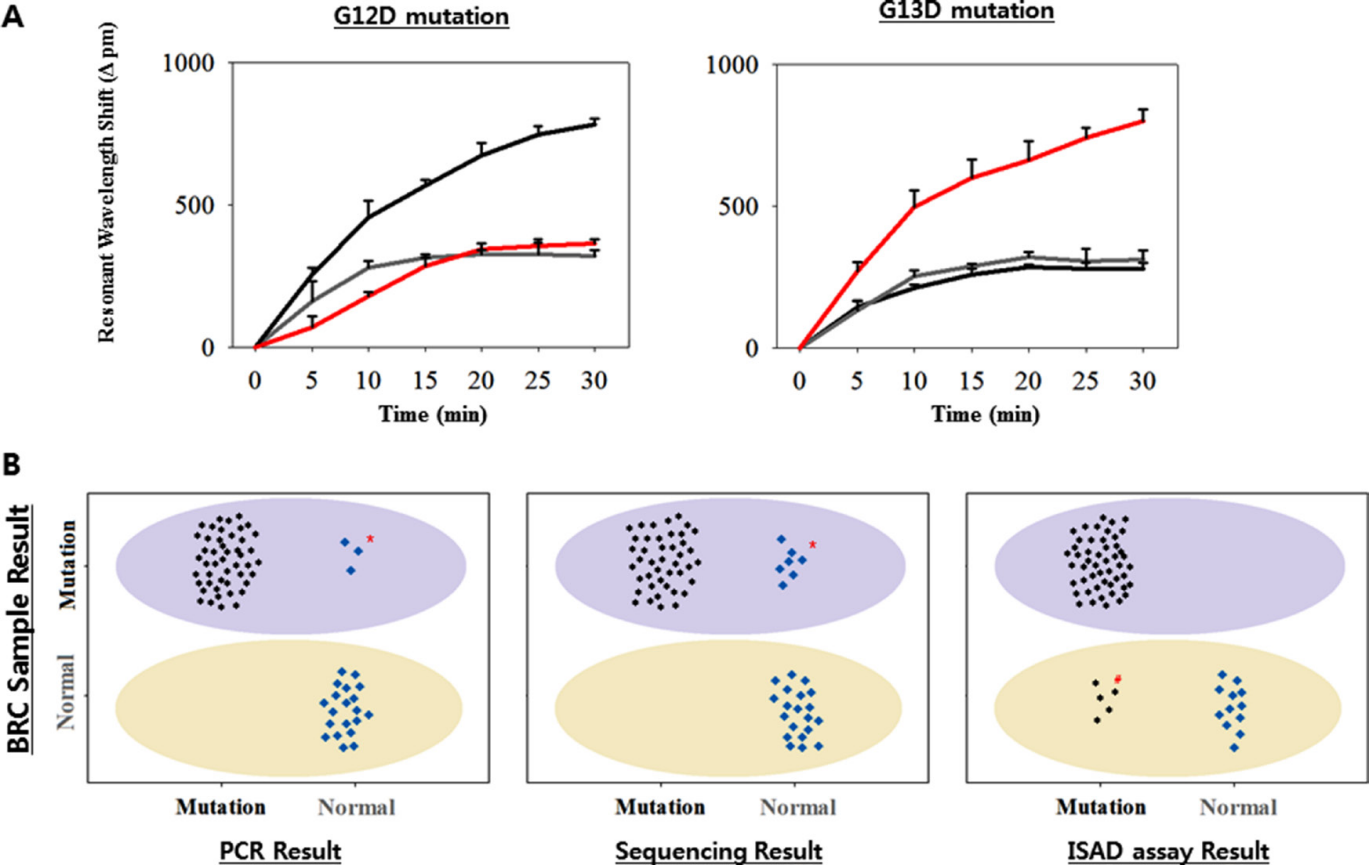
Validation of the ISAD-KRAS assay in 70 clinical samples (**A**) Representative wild and mutant cases from CRC sample. Resonant wavelength shift using the ISAD-KRAS assay with either the G12D mutant primer (left) or the G13D mutant primer (right). The colors represent wild-type sample (gray), G12D mutant sample (black), and G13D mutant sample (red). The error bars indicate the standard deviation of the mean, based on at least 3 independent experiments. (**B**) 50 mutants and 20 wild-type samples were screened by PCR (left), sequencing (center), and ISAD-KRAS (right). Based on the type of sample, as determined at the BRC, the light purple oval (mutant area) and yellow oval (wild-type area) areas were detected. The mutant allele (black) and wild allele (blue) are shown, based on the results of PCR, sequencing, and ISAD-KRAS assay. *, # indicate mis-matched results for the sample types between the 3 detection methods.

Similar to the G12D assay, when the G13D mutant primers were immobilized on the sensor, a resonant wavelength shift in the samples containing the G13D mutation of > 500 pm was considered as a positive result, whereas a resonant wavelength shift in the samples containing either no mutation or the G12D mutation of < 500 pm was considered as a negative result (Figure 4A). When the ISAD-KRAS assay was used for the 70 clinical samples, the G12D mutation was detected in 28 samples (40%) and the G13D mutation was detected in 27 samples (38.6%). The ISAD-KRAS assay was superior in the detection of mutations in the clinical samples, as compared to the reference values (Supplementary Table 2). In particular, 2 samples (no. 26, and 62 containing the G12D mutation) and 2 samples (no. 11, and 33 containing the G13D mutation) were identified using ISAD-KRAS and PCR. 2 samples (no. 24, and 32 containing the G12D mutation) and 1 sample (no. 59 containing the G13D mutation) were identified only using ISAD-KRAS, however PCR and sequencing could not identify the mutations in these samples as the number of mutant alleles within the samples may not have been sufficient (Supplementary Table 3). The genetic status of the *KRAS* mutations in the 70 CRC samples can be detected via PCR within 2–3 h, via sequencing within 1–2 days, and via the ISAD-KRAS assay within 30 min (Figure 1). Using the CRC samples, we examined the clinical sensitivity and specificity of these methods for the detection of both the G12D and G13D mutations (Table 1). When the 70 clinical samples were used to assess the sensitivity and specificity of PCR, 22 (sensitivity, 91.6%) were found to be positive for the G12D mutation, in comparison with 24 samples identified at the BRC; moreover, 48 samples (specificity, 100%) were found to be negative for the G12D mutation. Furthermore, 25 samples (sensitivity, 96.2%) were found to be positive for the G13D mutation, in comparison with the 26 samples identified at the BRC; in addition, 45 samples (specificity, 100%) were found to be negative for the G13D mutation (Table 1).

**Table 1:** Sensitivity and specificity of PCR, direct sequencing and ISAD-KRAS assay for *KRAS* mutation detection

Sample type	Positive sample					
	PCR result		Sequencing result		ISAD Result	
	Sensitivity	Specificity	Sensitivity	Specificity	Sensitivity	Specificity
G12D	91.6%	100%	87.5%	100%	100%	100%
G13D	96.2%	100%	84.6%	100%	100%	100%

Sensitivity: 100 X [TP/(TP + FN)], TP: true positive, FN: false negative.

Specificity: 100 X [TN/(FP + TN)], TN: true negative, FP: false positive.

When 70 clinical samples were used to assess the sensitivity and specificity of direct sequencing, 21 samples (sensitivity, 87.5%) were found to be positive for the G12D mutation, in comparison with the 24 samples identified at the BRC; moreover, 49 samples (specificity, 100%) were found to be negative for the G12D mutation. In addition, 22 samples (sensitivity, 84.6%) were found to be positive for the G13D mutation, in comparison with the 26 samples identified at the BRC; moreover, 48 samples (specificity, 100%) were found to be negative for the G13D mutation (Table 1 and Table 2). In contrast, the ISAD-KRAS assay showed a value of 100% for both the sensitivity and specificity with G12D and G13D mutations, when the 70 tumor specimens were examined (Table 1 and Supplementary Table 2). In particular, when 70 CRC samples were assessed using ISAD-KRAS, 5 cases (no. 13, 15, 18, and 68 for G12D and no. 5 for G13D) were found to have a mutation, which could not be detected by PCR and direct sequencing (Supplementary Table 3, Figure 4B).

**Table 2:** Clinicopathologic characteristics of the study patients according to colorectal cancer

Characteristics	Total No. (%)	*KRAS* wild type No. (%)	KRAS mutant			
			Codon 12		Codon 13	
			No. (%)	*p* value	No. (%)	*p* value
Total no. of patients	70	20	24		26	
Mean age (y) ± SD	58.5 (± 11.7)	60.8 (± 10.4)	58.5 (± 10.4)	0.173	56.5 (± 13.9)	0.169
Sex				0.651		0.446
Male	41 (58.6%)	13 (65%)	14 (58.3%)		14 (53.8%)	
Female	29 (41.4%)	7 (35%)	10 (41.7%)		12 (46.2%)	
CEA^a^ (ng/dl) ± SD	14.3 (± 1666.3)	5.7 (± 742.4)	29.3 (± 1051.4)	0.471	50.1 (± 2432.2)	0.43
Tumor location				0.710		0.869
Proximal	28 (40.0%)	7 (35%)	11 (45.8%)		10 (38.5%)	
Distal	19 (27.1%)	6 (30%)	7 (29.2%)		6 (23.1%)	
Rectum	23 (32.9%)	7 (35%)	6 (25%)		10 (38.5%)	
Borrmann type				0.531		0.512
I	3 (4.3%)	1 (5%)	2 (8.3%)		0 (0%)	
II	57 (81.4%)	17 (85%)	17 (70.8%)		23 (88.5%)	
III	10 (14.3%)	2 (10%)	5 (20.8%)		3 (11.5%)	
Disease stage				0.106		0.063
II	4 (5.7%)	3 (%)	2 (8.3%)		0 (0%)	
III	13 (18.6%)	6 (%)	2 (8.3%)		5 (19.2%)	
IV	52 (74.3%)	11 (55%)	20 (83.3%)		21 (80.8%)	
Cell type				0.586		0.474
ADC^b^_WD	1 (1.4%)	1 (5%)	0 (0%)		0 (0%)	
ADC_MD	60 (85.7%)	17 (85%)	22 (91.7%)		20 (76.9%)	
ADC_PD	5 (7.1%)	2 (10%)	1 (4.2%)		2 (7.7%)	
Mucinous	4 (5.7%)	0 (0%)	1 (4.2%)		4 (15.4%)	
MSI^c^ status				0.350		0.473
MSS	56 (80%)	17 (85%)	20 (83.3%)		19 (73.1%)	
MSI low	3 (4.3%)	0 (0%)	2 (8.3%)		1 (3.8%)	
MSI high	2 (2.9%)	0 (0%)	0 (0%)		2 (7.7%)	
LVI^d^	51 (72.9%)	18 (72%)	18 (75%)	0.711	19 (73.1%)	0.818
PNI^e^	49 (70.0%)	20 (80%)	20 (83.3%)	0.162	16 (61.5%)	0.809
IHC^f^						
hMLH1	69 (98.6%)	20 (100%)	23 (95.8%)	0.356	26 (100%)	>.05
hMLH2	68 (97.1%)	19 (95)	23 (95.8%)	0.362	26 (100%)	0.249
p53				0.566		0.512
negative	15 (21.4%)	4 (20%)	4 (16.7%)		7 (26.9%)	
positive	54 (77.1%)	15 (75%)	20 (83.3%)		19 (73.1%)	
CEA_Density				0.087		0.364
1+	4 (5.7%)	0 (0%)	2 (8.3%)		2 (7.7%)	
2+	14 (20.0%)	2 (10%)	8 (33.3%)		4 (15.4%)	
3+	51 (72.9%)	17 (85%)	14 (58.3%)		20 (76.9%)	
CEA_Pattern				0.598		0.525
Apico	25 (35.7%)	5 (25%)	15 (62.5%)		18 (69.2%)	
Diffuse	12 (17.1%)	2 (10%)	4 (16.7%)		6 (23.1%)	
Mixed	13 (18.6%)	3 (15%)	5 (20.8%)		2 (7.7%)	
Gene mutation						
*BRAF*	0 (0%)	0 (0%)	0 (0%)	0.268	0 (0%)	0.849
EGFR	63 (90.0%)	16 (80%)	24 (100%)	0.071	23 (88.5%)	0.710

^a^CEA : carcinoembryonic antigen.

^b^ADC : adenocarcinoma/WD : well-differentiated/MD : moderately differentiated/PD : poorly-differentiated.

^c^MSI : microsatellite instability.

^d^LVI : lymphovascular invasion.

^e^PNI : perineural invasion.

^f^IHC : immunohistochemistry.

PCR and direct sequencing were unable to detect 3 and 7 samples containing *KRAS* mutations, respectively; the presence of *KRAS* mutations in these samples was confirmed by sequencing at the BRC. This may be due to the difference in the locations of the CRC samples from which DNA was extracted. This result is consistent with several studies that showed that the DNA from cancer samples isolated from different regions shows a change in the copy number, with very few discrepancies [50–52]. Nevertheless, we could identify the *KRAS* mutations in these samples by using the ISAD-KRAS assay. In particular, ISAD-KRAS detected the mutations from 5 wild-type samples, including 4 samples with the G12D mutation and 1 sample with the G13D mutation (Figure 4, right, Supplementary Table 3); these mutations could not be detected using PCR and direct sequencing. To confirm the ISAD-KRAS results, we assessed several issues. First, we re-extracted the DNA from these samples to perform ISAD-KRAS once more, using the new samples; however, the results obtained were similar. Second, we assessed the treatment history of the patient to confirm the response of the drug based on the *KRAS* mutation status. However, we could not predict the presence of mutations in the patients as they did not receive anti-EGFR drugs for their treatment. Although there is no other method for confirming the mutations identified by ISAD-KRAS, we found that the performance of the ISAD-KRAS assay in the present study is highly sensitive and specific. Thus, the clinical sensitivity and specificity of the ISAD-KRAS assay in clinical samples is superior to those of the conventional methods.

## DISCUSSION

Given that many clinically important cancer-related mutations and genes have been identified thus far, the need for a fast and simple diagnostic assay has become urgent, in order to prescribe effective cancer treatment before or after surgery. Methods such as PCR, and PCR-related technologies, are relatively accurate, sensitive and convenient compared to the direct sequencing protocols that have been developed for clinical use. However, these methods remain time-consuming (> 3 h), involve complicated steps (separate amplification and detection), and require the use of a thermal cycler. Therefore, these applications are not suitable for routine examination and are subsequently difficult to implement in the hospital. In the present study, we report the use of a rapid and accurate assay as a companion diagnostic tool for the analysis of G12D and G13D mutations of the *KRAS* gene in CRC samples, which can provide better information for anti-EGFR drug treatment (Figure 1). The ISAD-KRAS assay involves the use of the SMR optical sensor, which enables label-free and rapid detection, as well as the isothermal DNA amplification method, which can simultaneously amplify and detect mutations from the clinical samples. A limitation of the ISAD-KRAS assay is that it can only be used to detect known mutations, as the target can then be identified by the complementary sequence of target primers on the sensor surface. Therefore, if there are non-identical mutations in the templates, novel mutations may be overlooked with this technology. Hence, the ISAD-KRAS assay is suitable for determining the status of known mutations, such as G12D and G13D, in clinical CRC samples, as a companion diagnostic tool prior to making the treatment decision. Furthermore, this assay can be useful for detecting other hot-spot mutations in clinical specimens such as stool and blood by changing the primer of target gene on the sensor surface. Such a system will further optimize the protocol with a large cohort for the improvement of the versatility and utility in clinical applications. We believe that ISAD-KRAS has significant potential as a high-resolution diagnostic tool for the selection of candidate patients, and should enable more personalized therapies in human cancer applications.

## MATERIALS AND METHODS

### Principle and operation of the ISAD-KRAS assay

The ISAD assay is an optical sensor-based isothermal nucleic acid amplification technique that can rapidly detect single point mutations within 30 min [39]. Briefly, one part of the primer is covalently immobilized on the sensor surface to start DNA synthesis. Thereafter, a mixture of the other part of the primer, the target genomic DNA, and RPA is added to the SMR surface. The amplification process of the ISAD-KRAS assay is initiated by the binding of the recombinase-primer complexes to double-stranded DNA, facilitating strand exchange. DNA amplification is achieved by the polymerase, thus exponentially increasing the number of DNA copies. Finally, the DNA that has been exponentially amplified through the repetition of the process on the sensor surface, is monitored via a wavelength shift on the optical sensor in a label-free and real-time manner. Following the amplification of the target DNA with the immobilized primer on the SMR surface, the wavelength shift occurs as the light is concentrated at the surface in a certain area due to the evanescent field effect [36]. The assay is performed at 38°C for 30 min.

### Preparation of the ISAD-KRAS assay

The detailed preparation of the SMRs for the ISAD-KRAS assay was the previously described protocol [39, 42]. Briefly, the sensors were treated with oxygen plasma for the purpose of cleaning and surface oxidation, and were then immersed in a solution of 2% 3-aminopropyltriethoxysilane (APTES) in a mixture of ethanol–H_2_O (95%: 5%, v/v) for 2 h, followed by thorough rinsing with ethanol and de-ionized (DI) water. The sensors were then incubated with 2.5% glutaraldehyde (GAD) in DI water containing 5 mM sodium cyanoborohydride for 1 h, followed by rinsing with DI water. The chemical reagents, including APTES, GAD solution (50% weight in water), and sodium cyanoborohydride solution (5.0 M in 1 M NaOH), were purchased from Sigma-Aldrich (St. Louis, MO). All the primers used for the RPA and conventional PCR assays, for the amplification of the *KRAS* gene, are shown in Supplementary Table 1, and were custom-synthesized (Macrogen Inc. Seoul, Korea). For DNA primer immobilization, the sensor surface was prepared by incubation with the *KRAS* primer in PBS (1 mM) containing 5 mM sodium cyanoborohydride for 16 h at room temperature. The optimal concentration of primer for grafting on the sensor was used as the previously reported [35, 39]. Following incubation, unbound DNA probes were washed away with PBS, and the sensors were dried using nitrogen. An acrylic well was then pasted onto the chip to enclose the optical sensor area. The RPA solution was then prepared for the amplification and detection of target DNA with the ISAD-KRAS assay. For an optimized reaction in clinical samples, 29.5 μL of rehydration buffer, 15 μL of nuclease-free water, and 2.5 μL of forward primers (10 μM) were combined. One dried enzyme pellet (TwistDX, UK) was added to each solution and vortexed. Thereafter, 2.5 μL of magnesium acetate solution (280 mM) was dispensed into the cap of each tube. A unidirectional shake mode mixing protocol guaranteed the homogeneous distribution of the molecules necessary for the reaction, in the buffer. After mixing, 50 μL of reaction buffer was split into 5 μL aliquots. To initiate the reactions, 5 μL of DNA extracted from cancer cell lines and tissues from CRC patients, were added to each 5 μL reaction aliquot. Finally, we added the RPA solutions containing the DNA targets to the acrylic well at room temperature. We then added mineral oil to protect the solution from evaporation during amplification and detection. The ISAD-KRAS assay mixture was then placed on a thermo electric cooler (TEC), with a controller (Alpha Omega Instruments) to maintain a constant temperature (38°C) through the application of a constant DC voltage. The resonance spectrum of the device was immediately measured and used as a reference to obtain a baseline value.

### Cancer cell lines

The AGS stomach cancer cell line (ATCC_CRL-1739), which contains the G12D point mutation (codon 12 GGT→GAT), and the HCT116 colorectal cancer cell line (ATCC_CCL-247), which contains the G13D point mutation (codon 13 GGC→GAC), were maintained in plastic culture dishes with high-glucose Dulbecco`s Modified Eagle`s Medium (DMEM, Life Technology), supplemented with 10% fetal bovine serum (FBS) in a 37°C humid incubator with 5% ambient CO_2_. To compare the limit of detection (LOD) for PCR, direct sequencing, and ISAD-KRAS assays, the AGS and HCT116 cell lines were serially mixed, after which the DNA was extracted using the QIAamp DNA mini kit (Qiagen, Germany). The cell mixtures for the detection of the G12D mutation were prepared by mixing them with the AGS (G12D mutant cells) and HCT116 (wild-type cells) cell lines at the desired percentage, ranging from 0%, 1%, 10%, 30%, and 50% to 100% of mutant cells. Moreover, the cell mixtures for the detection of the G13D mutation were prepared by mixing them with the HCT116 (G13D mutant cell lines) and AGS (wild-type) cell lines at the desired percentage, ranging from 0%, 1%, 10%, 30%, and 50% to 100% of mutant cells. The serially diluted samples with either G12D or G13D were eluted with 100 μL of elution buffer. The eluted DNA was stored at –20°C until use.

### PCR and sequencing analysis

The forward and reverse primers for the amplification of the *KRAS* mutations (G12D and G13D) were synthesized at the usual length of approximately 24 bps (Supplementary Table 1). The end-point PCR process comprised an initial denaturation step at 95°C for 15 min, followed by 35 cycles at 95°C for 30 s, 59°C for 30 s, and 72°C for 30 s, as well as a final elongation step at 72°C for 7 min. Moreover, 5 μL of DNA was amplified in a total volume of 25 μL, containing 10X PCR buffer (Qiagen), 2.5 mM MgCl_2_, 0.25 mM deoxynucleotide triphosphate, 25 pmol of each primer, and 1 unit of Taq DNA polymerase (Qiagen). Gel electrophoresis was used to separate the PCR products on a 2% agarose gel containing ethidium bromide (EtBr). The gel was visualized using a GelDoc System (Clinx Science Instruments). For direct sequencing of DNA, all the DNA samples were amplified (annealing temperature of 55°C) with the sequencing primer of *KRAS* exon 2, and were then purified by using the Expin PCR SV (GeneAll, Korea). The purified samples were directly sequenced using BigDyeTerminal chemistry, with the forward sequencing primer. The DNA sequencing reaction mixtures were electrophoresed using ABI's 3730XL DNA analyzer (Applied Biosystems, USA) at the Macrogen sequencing center (Macrogen Inc. Seoul, Korea).

### Clinical sample collection

A total of 70 cancer samples (24 samples with G12D mutations, 26 samples with G13D mutation, and 20 samples with no mutation in exon 2) based on frozen tissue availability were obtained from the BRC of Asan Medical Center (Seoul, Korea), after approval from the Institutional Review Board (IRB_Nr. 2016–0809). The samples had been obtained by a colorectal surgical team, and were randomly selected according to the type of mutation (Table 2). The clinicopathologic characteristics of all the patients, which are known to be related to the course of CRCs were obtained from a prospectively collected database (Table 2). The characteristics of all the patients in the present study are described in Table 1. The mean age of the CRC patients was 58.5 years, and 41 patients (58.6%) were male. Stage I, II, III, and IV tumors were observed in 0 (0%), 4 (5.7%), 13 (18.6%), and 52 (74.3%) patients, respectively. Among the 50 patients with the *KRAS* mutation and without *BRAF* mutations, 24 had the G12D mutation (codon 12 GGTèGAT) and the remaining 26 had the G13D mutation (codon 13 GGCèGAC). The types of CRC tissue samples were identified by direct sequencing at BRC, and were used as references. The genomic DNAs from the tissues were extracted using ATL buffer with proteinase K from a QIAamp DNA mini kit (Qiagen, Germany) according to the manufacturer's instructions. The samples were eluted using 100 μL of elution buffer. The eluted DNA was stored at –20°C until use.

## SUPPLEMENTARY MATERIALS FIGURES AND TABLES





## References

[1] Walther A, Johnstone E, Swanton C, Midgley R, Tomlinson I, Kerr D (2009). Genetic prognostic and predictive markers in colorectal cancer. Nat Rev Cancer.

[2] Chiu HM, Chang LC, Hsu WF, Chou CK, Wu MS (2015). Non-invasive screening for colorectal cancer in Asia. Best Pract Res Clin Gastroenterol.

[3] Molinari F, Felicioni L, Buscarino M, De Dosso S, Buttitta F, Malatesta S, Movilia A, Luoni M, Boldorini R, Alabiso O, Girlando S, Soini B, Spitale A (2011). Increased detection sensitivity for KRAS mutations enhances the prediction of anti-EGFR monoclonal antibody resistance in metastatic colorectal cancer. Clin Cancer Res.

[4] Imamura Y, Morikawa T, Liao X, Lochhead P, Kuchiba A, Yamauchi M, Qian ZR, Nishihara R, Meyerhardt JA, Haigis KM, Fuchs CS, Ogino S (2012). Specific mutations in KRAS codons 12 and 13, and patient prognosis in 1075 BRAF wild-type colorectal cancers. Clin Cancer Res.

[5] Yoon HH, Tougeron D, Shi Q, Alberts SR, Mahoney MR, Nelson GD, Nair SG, Thibodeau SN, Goldberg RM, Sargent DJ, Sinicrope FA, and Alliance for Clinical Trials in Oncology (2014). KRAS codon 12 and 13 mutations in relation to disease-free survival in BRAF-wild-type stage III colon cancers from an adjuvant chemotherapy trial (N0147 alliance). Clin Cancer Res.

[6] Tougeron D, Lecomte T, Pagès JC, Villalva C, Collin C, Ferru A, Tourani JM, Silvain C, Levillain P, Karayan-Tapon L (2013). Effect of low-frequency KRAS mutations on the response to anti-EGFR therapy in metastatic colorectal cancer. Ann Oncol.

[7] Laurent-Puig P, Pekin D, Normand C, Kotsopoulos SK, Nizard P, Perez-Toralla K, Rowell R, Olson J, Srinivasan P, Le Corre D, Hor T, El Harrak Z, Li X (2015). Clinical relevance of KRAS-mutated subclones detected with picodroplet digital PCR in advanced colorectal cancer treated with anti-EGFR therapy. Clin Cancer Res.

[8] Sun C, Hobor S, Bertotti A, Zecchin D, Huang S, Galimi F, Cottino F, Prahallad A, Grernrum W, Tzani A, Schlicker A, Wessels LF, Smit EF (2014). Intrinsic resistance to MEK inhibition in KRAS mutant lung and colon cancer through transcriptional induction of ERBB3. Cell Reports.

[9] Prenen H, Tejpar S, Van Cutsem E (2010). New strategies for treatment of KRAS mutant metastatic colorectal cancer. Clin Cancer Res.

[10] Imamura Y, Lochhead P, Yamauchi M, Kuchiba A, Qian ZR, Liao X, Nishihara R, Jung S, Wu K, Nosho K, Wang YE, Peng S, Bass AJ (2014). Analyses of clinicopathological, molecular, and prognostic associations of KRAS codon 61 and codon 146 mutations in colorectal cancer: cohort study and literature review. Mol Cancer.

[11] Phipps AI, Buchanan DD, Makar KW, Win AK, Baron JA, Lindor NM, Potter JD, Newcomb PA (2013). KRAS-mutation status in relation to colorectal cancer survival: the joint impact of correlated tumour markers. Br J Cancer.

[12] Misale S, Yaeger R, Hobor S, Scala E, Janakiraman M, Liska D, Valtorta E, Schiavo R, Buscarino M, Siravegna G, Bencardino K, Cercek A, Chen CT (2012). Emergence of KRAS mutations and acquired resistance to anti-EGFR therapy in colorectal cancer. Nature.

[13] Petrelli F, Borgonovo K, Cabiddu M, Ghilardi M, Barni S (2011). Cetuximab and panitumumab in KRAS wild-type colorectal cancer: a meta-analysis. Int J Colorectal Dis.

[14] Rosty C, Young JP, Walsh MD, Clendenning M, Walters RJ, Pearson S, Pavluk E, Nagler B, Pakenas D, Jass JR, Jenkins MA, Win AK, Southey MC (2013). Colorectal carcinomas with KRAS mutation are associated with distinctive morphological and molecular features. Mod Pathol.

[15] Huang JF, Zeng DZ, Duan GJ, Shi Y, Deng GH, Xia H, Xu HQ, Zhao N, Fu WL, Huang Q (2015). Single-tubed wild-type blocking quantitative PCR detection assay for the sensitive detection of codon 12 and 13 KRAS mutations. PLoS One.

[16] Kimura T, Okamoto K, Miyamoto H, Kimura M, Kitamura S, Takenaka H, Muguruma N, Okahisa T, Aoyagi E, Kajimoto M, Tsuji Y, Kogawa T, Tsuji A, Takayama T (2012). Clinical benefit of high-sensitivity KRAS mutation testing in metastatic colorectal cancer treated with anti-EGFR antibody therapy. Oncology.

[17] Jimeno A, Messersmith WA, Hirsch FR, Franklin WA, Eckhardt SG (2009). KRAS mutations and sensitivity to epidermal growth factor receptor inhibitors in colorectal cancer: practical application of patient selection. J Clin Oncol.

[18] Tsiatis AC, Norris-Kirby A, Rich RG, Hafez MJ, Gocke CD, Eshleman JR, Murphy KM (2010). Comparison of Sanger sequencing, pyrosequencing, and melting curve analysis for the detection of KRAS mutations: diagnostic and clinical implications. J Mol Diagn.

[19] Carotenuto P, Roma C, Rachiglio AM, Tatangelo F, Pinto C, Ciardiello F, Nappi O, Iaffaioli RV, Botti G, Normanno N (2010). Detection of KRAS mutations in colorectal carcinoma patients with an integrated PCR/sequencing and real-time PCR approach. Pharmacogenomics.

[20] Angulo B, García-García E, Martínez R, Suárez-Gauthier A, Conde E, Hidalgo M, López-Ríos F (2010). A commercial real-time PCR kit provides greater sensitivity than direct sequencing to detect KRAS mutations: a morphology-based approach in colorectal carcinoma. J Mol Diagn.

[21] Dufort S, Richard MJ, de Fraipont F (2009). Pyrosequencing method to detect KRAS mutation in formalin-fixed and paraffin-embedded tumor tissues. Anal Biochem.

[22] Pekin D, Skhiri Y, Baret JC, Le Corre D, Mazutis L, Salem CB, Millot F, El Harrak A, Hutchison JB, Larson JW, Link DR, Laurent-Puig P, Griffiths AD, Taly V (2011). Quantitative and sensitive detection of rare mutations using droplet-based microfluidics. Lab Chip.

[23] Harlé A, Busser B, Rouyer M, Harter V, Genin P, Leroux A, Merlin JL (2013). Comparison of COBAS 4800 KRAS, TaqMan PCR and high resolution melting PCR assays for the detection of KRAS somatic mutations in formalin-fixed paraffin embedded colorectal carcinomas. Virchows Arch.

[24] Lee S, Brophy VH, Cao J, Velez M, Hoeppner C, Soviero S, Lawrence HJ (2012). Analytical performance of a PCR assay for the detection of KRAS mutations (codons 12/13 and 61) in formalin-fixed paraffin-embedded tissue samples of colorectal carcinoma. Virchows Arch.

[25] Chang YS, Yeh KT, Chang TJ, Chai C, Lu HC, Hsu NC, Chang JG (2009). Fast simultaneous detection of K-RAS mutations in colorectal cancer. BMC Cancer.

[26] Huggett JF, Cowen S, Foy CA (2015). Considerations for digital PCR as an accurate molecular diagnostic tool. Clin Chem.

[27] Simi L, Pratesi N, Vignoli M, Sestini R, Cianchi F, Valanzano R, Nobili S, Mini E, Pazzagli M, Orlando C (2008). High-resolution melting analysis for rapid detection of KRAS, BRAF, and PIK3CA gene mutations in colorectal cancer. Am J Clin Pathol.

[28] Franklin WA, Haney J, Sugita M, Bemis L, Jimeno A, Messersmith WA (2010). KRAS mutation: comparison of testing methods and tissue sampling techniques in colon cancer. J Mol Diagn.

[29] Taly V, Pekin D, Benhaim L, Kotsopoulos SK, Le Corre D, Li X, Atochin I, Link DR, Griffiths AD, Pallier K, Blons H, Bouché O, Landi B (2013). Multiplex picodroplet digital PCR to detect KRAS mutations in circulating DNA from the plasma of colorectal cancer patients. Clin Chem.

[30] Dressman D, Yan H, Traverso G, Kinzler KW, Vogelstein B (2003). Transforming single DNA molecules into fluorescent magnetic particles for detection and enumeration of genetic variations. Proc Natl Acad Sci USA.

[31] Thierry AR, Mouliere F, El Messaoudi S, Mollevi C, Lopez-Crapez E, Rolet F, Gillet B, Gongora C, Dechelotte P, Robert B, Del Rio M, Lamy PJ, Bibeau F (2014). Clinical validation of the detection of KRAS and BRAF mutations from circulating tumor DNA. Nat Med.

[32] Jayanthi VS, Das AB, Saxena U (2017). Recent advances in biosensor development for the detection of cancer biomarkers. Biosens Bioelectron.

[33] Wei F, Patel P, Liao W, Chaudhry K, Zhang L, Arellano-Garcia M, Hu S, Elashoff D, Zhou H, Shukla S, Shah F, Ho CM, Wong DT (2009). Electrochemical sensor for multiplex biomarkers detection. Clin Cancer Res.

[34] Liu Q, Nam J, Kim S, Lim CT, Park MK, Shin Y (2016). Two-stage sample-to-answer system based on nucleic acid amplification approach for detection of malaria parasites. Biosens Bioelectron.

[35] Park MK, Kee JS, Quah JY, Netto V, Song J, Fang Q, La Fosse EM, Lo GQ (2013). Label-free aptamer sensor based on silicon microring resonators. Sens Actuators B Chem.

[36] Bogaerts W, De Heyn P, Van Vaerenbergh T, De Vos K, Slevaraja SK, Claes T, Dumon P, Bienstman P, Van Thourhout D, Baets R (2012). Silicon Microring Resonators. Laser Photonics Rev.

[37] Shin Y, Perera AP, Kee JS, Song J, Fang Q, Lo GQ, Park MK (2013). Label-free methylation specific sensor based on silicon microring resonators for detection and quantification of DNA methylation biomarkers in bladder cancer. Sens Actuators B Chem.

[38] Iqbal M, Gleeson MA, Spaugh B, Tybor F, Gunn WG, Hochberg M, Baehr-Jones MT, Bailey RC, Gunn LC (2010). Label-free biosensor arrays based on silicon ring resonators and high-speed optical scanning instrumentation. IEEE J Sel Top Quantum Electron.

[39] Shin Y, Perera AP, Kim KW, Park MK (2013). Real-time, label-free isothermal solid-phase amplification/detection (ISAD) device for rapid detection of genetic alteration in cancers. Lab Chip.

[40] Koo B, Jin CE, Lee TY, Lee JH, Park MK, Sung H, Park SY, Lee HJ, Kim SM, Kim JY, Kim SH, Shin Y (2017). An isothermal, label-free, and rapid one-step RNA amplification/detection assay for diagnosis of respiratory viral infections. Biosens Bioelectron.

[41] Kim KW, Shin Y, Perera AP, Liu Q, Kee JS, Han K, Yoon YJ, Park MK (2013). Label-free, PCR-free chip-based detection of telomerase activity in bladder cancer cells. Biosens Bioelectron.

[42] Shin Y, Perera AP, Tang WY, Fu DL, Liu Q, Sheng JK, Gu Z, Lee TY, Barkham T, Park MK (2015). A rapid amplification/detection assay for analysis of Mycobacterium tuberculosis using an isothermal and silicon bio-photonic sensor complex. Biosens Bioelectron.

[43] Zeng S, Baillargeat D, Ho HP, Yong KT (2014). Nanomaterials enhanced surface plasmon resonance for biological and chemical sensing applications. Chem Soc Rev.

[44] Guo X (2012). Surface plasmon resonance based biosensor technique: a review. J Biophotonics.

[45] Piepenburg O, Williams CH, Stemple DL, Armes NA (2006). DNA detection using recombination proteins. PLoS Biol.

[46] Lutz S, Weber P, Focke M, Faltin B, Hoffmann J, Müller C, Mark D, Roth G, Munday P, Armes N, Piepenburg O, Zengerle R, von Stetten F (2010). Microfluidic lab-on-a-foil for nucleic acid analysis based on isothermal recombinase polymerase amplification (RPA). Lab Chip.

[47] Shen F, Davydova EK, Du W, Kreutz JE, Piepenburg O, Ismagilov RF (2011). Digital isothermal quantification of nucleic acids via simultaneous chemical initiation of recombinase polymerase amplification reactions on SlipChip. Anal Chem.

[48] Euler M, Wang Y, Nentwich O, Piepenburg O, Hufert FT, Weidmann M (2012). Recombinase polymerase amplification assay for rapid detection of Rift Valley fever virus. J Clin Virol.

[49] Rohrman BA, Richards-Kortum RR (2012). A paper and plastic device for performing recombinase polymerase amplification of HIV DNA. Lab Chip.

[50] Stransky N, Vallot C, Reyal F, Bernard-Pierrot I, de Medina SG, Segraves R, de Rycke Y, Elvin P, Cassidy A, Spraggon C, Graham A, Southgate J, Asselain B (2006). Regional copy number-independent deregulation of transcription in cancer. Nat Genet.

[51] Midorikawa Y, Tsutsumi S, Nishimura K, Kamimura N, Kano M, Sakamoto H, Makuuchi M, Aburatani H (2004). Distinct chromosomal bias of gene expression signatures in the progression of hepatocellular carcinoma. Cancer Res.

[52] Furge KA, Dykema KJ, Ho C, Chen X (2005). Comparison of array-based comparative genomic hybridization with gene expression-based regional expression biases to identify genetic abnormalities in hepatocellular carcinoma. BMC Genomics.

