# Supplementary material for: Rapid and accurate detection of KRAS mutations in colorectal cancers using the isothermal-based optical sensor for companion diagnostics
Source: Oncotarget. 2017 Aug 8;8(48):83860–71. doi: 10.18632/oncotarget.20038 (PMC5663560; doi:10.18632/oncotarget.20038)
Supplement: Supplementary file 1 [file oncotarget-08-83860-s001.pdf]

# Rapid and accurate detection of *KRAS* mutations in colorectal cancers using the isothermal-based optical sensor for companion diagnostics

## SUPPLEMENTARY MATERIALS

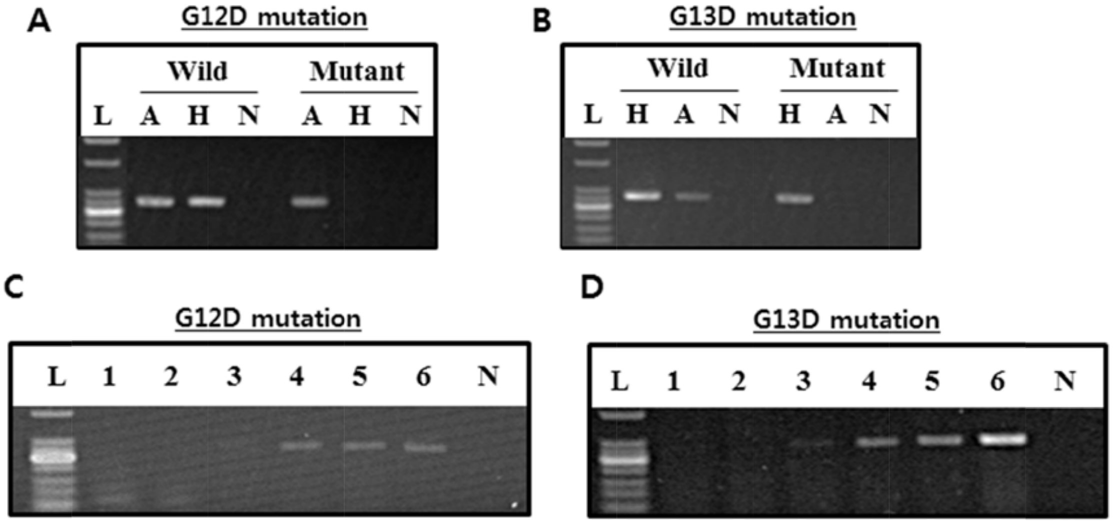


**Supplementary Figure 1: Characterization of *KRAS* mutant primers using PCR.** (**A–B**) Gel electrophoresis after PCR with the wild-type primer and G12D mutant primer (A; L: 50 bp DNA marker, A: DNA obtained from the AGS cell line, H: DNA obtained from the HCT116 cell line, N: no DNA). DNA amplification with the wild-type primer and G13D mutant primer (B; L: 50 bp DNA marker, A: DNA obtained from the AGS cell line, H: DNA obtained from the HCT116 cell line, N: no DNA). (C-D), Gel electrophoresis after PCR with the G12D mutant primer (**C**) and the G13D mutant primer (**D**) (L: 50 bp DNA marker, 1: 0%, 2: 1%, 3: 10%, 4: 30%, 5: 50%, 6: 100% of mutant cells, N: no DNA).

**Supplementary Table 1: Sequences of the primers used for PCR, Sequencing and ISAD-KRAS assay**

| *Target* | Type | | Sequence |
|---|---|---|---|
| G12D | PCR (Wild) | Forward | 5′-TGTGGTAGTTGGAGCTGG-3′ |
| | | Reverse | 5′-TCATGAAAATGGTCAGAGAAACC-3′ |
| | PCR (Mutation) | Forward | 5′-TGTGGTAGTTGGAGCTGA-3′ |
| | | Reverse | 5′-TCATGAAAATGGTCAGAGAAACC-3′ |
| | ISAD | Forward | 5′-NH2-(CH2)12-CTGAATATAAACTTGTGGTAGTTGGAGCTGA-3′ |
| | | Reverse | 5′-NH2-(CH2)12-CTCATGAAAATGGTCAGAGAAACCTTTATC-3′ |
| G13D | PCR (Wild) | Forward | 5′-TGTGGTAGTTGGAGCTGGTGG-3′ |
| | | Reverse | 5′-TCATGAAAATGGTCAGAGAAACC-3′ |
| | PCR (Mutation) | Forward | 5′-TGTGGTAGTTGGAGCTGGTGAG-3′ |
| | | Reverse | 5′-TCATGAAAATGGTCAGAGAAACC-3′ |
| | ISAD | Forward | 5′-NH$_2$-(CH2)$_{12}$-CTGAATATAAACTTGTGGTAGTTGGAGCTGGTGAG-3′ |
| | | Reverse | 5′-NH$_2$-(CH2)$_{12}$-CTCATGAAAATGGTCAGAGAAACCTTTATC-3′ |
| Exon 2 | Sequencing | Forward | 5′-TCATTATTTTTATTATAAGGCCTGCT-3′ |
| | | Reverse | 5′-CAAGATTTACCTCTATTGTTGGATC-3′ |

**Supplementary Table 2: Accuracy of *KRAS* mutation detection in 70 clinical samples using PCR, direct sequencing and ISAD-KRAS assay**

| Sample (reference) | Total tested sample | Positive sample | | | | | |
|---|---|---|---|---|---|---|---|
| | | PCR result | | Sequencing result | | ISAD Result | |
| | | G12D | G13D | G12D | G13D | G12D | G13D |
| Wild | 20 | 0 | 0 | 0 | 0 | 4* | 1* |
| G12D | 24 | 22 | 0 | 21 | 0 | 24 | 0 |
| G13D | 26 | 0 | 25 | 0 | 22 | 0 | 26 |
| Total | 70 | 22/70 (31.4%) | 25/70 (35.7%) | 21/70 (30%) | 22/70 (31.4%) | 28/70 (40%) | 27/70 (38.6%) |

* Newly detected mutations in wild-type samples using ISAD.

**Supplementary Table 3: Results of end-point PCR, direct sequencing and ISAD-KRAS assays in 70 clinical samples.** See Supplementary_Table_3
