# Supplementary material for: Rapid and accurate detection of KRAS mutations in colorectal cancers using the isothermal-based optical sensor for companion diagnostics
Source: Oncotarget. 2017 Aug 8;8(48):83860–71. doi: 10.18632/oncotarget.20038 (PMC5663560; doi:10.18632/oncotarget.20038)
Supplement: Supplementary file 2 [file oncotarget-08-83860-s002.docx]

**Supplementary Table 3.** Results of end-point PCR, direct sequencing and ISAD-KRAS assays in 70 clinical samples

|  | BRC | End-point PCR | | Direct  Sequencing | ISAD | |
| --- | --- | --- | --- | --- | --- | --- |
| No. | Sample type | G12D | G13D |  | G12D | G13D |
| 1 | G13D | X | O | G13D | X | O |
| 2 | Wild | X | X | Wild | X | X |
| 3 | Wild | X | X | Wild | X | X |
| 4 | Wild | X | X | Wild | X | X |
| 5 | Wild | X | X | Wild | X | O |
| 6 | Wild | X | X | Wild | X | X |
| 7 | G13D | X | O | G13D | X | O |
| 8 | Wild | X | X | Wild | X | X |
| 9 | Wild | X | X | Wild | X | X |
| 10 | Wild | X | X | Wild | X | X |
| 11 | G13D | X | O | Wild | X | O |
| 12 | Wild | X | X | Wild | X | X |
| 13 | Wild | X | X | Wild | O | X |
| 14 | Wild | X | X | Wild | X | X |
| 15 | Wild | X | X | Wild | O | X |
| 16 | G13D | X | O | G13D | X | O |
| 17 | Wild | X | X | Wild | X | X |
| 18 | Wild | X | X | Wild | O | X |
| 19 | G12D | O | X | G12D | O | X |
| 20 | Wild | X | X | Wild | X | X |
| 21 | G13D | X | O | G13D | X | O |
| 22 | G13D | X | O | G13D | X | O |
| 23 | G12D | O | X | G12D | O | X |
| 24 | G12D | X | X | Wild | O | X |
| 25 | G13D | X | O | G13D | X | O |
| 26 | G13D | X | O | Wild | X | O |
| 27 | G12D | O | X | G12D | O | X |
| 28 | G12D | O | X | G12D | O | X |
| 29 | Wild | X | X | Wild | X | X |
| 30 | G13D | X | O | G13D | X | O |
| 31 | G12D | O | X | G12D | O | X |
| 32 | G12D | X | X | Wild | O | X |
| 33 | G13D | X | O | Wild | X | O |
| 34 | G13D | X | O | G13D | X | O |
| 35 | G12D | O | X | G12D | O | X |
| 36 | G13D | X | O | G13D | X | O |
| 37 | G12D | O | X | G12D | O | X |
| 38 | G12D | O | X | G12D | O | X |
| 39 | G13D | X | O | G13D | X | O |
| 40 | G13D | X | O | G13D | X | O |
| 41 | G12D | O | X | G12D | O | X |
| 42 | G12D | O | X | G12D | O | X |
| 43 | Wild | X | X | Wild | X | X |
| 44 | G13D | X | O | G13D | X | O |
| 45 | Wild | X | X | Wild | X | X |
| 46 | G13D | X | O | G13D | X | O |
| 47 | Wild | X | X | Wild | X | X |
| 48 | G12D | O | X | G12D | O | X |
| 49 | G12D | O | X | G12D | O | X |
| 50 | G12D | O | X | G12D | O | X |
| 51 | G12D | O | X | G12D | O | X |
| 52 | G13D | X | O | G13D | X | O |
| 53 | G12D | O | X | G12D | O | X |
| 54 | G13D | X | O | G13D | X | O |
| 55 | G12D | O | X | G12D | O | X |
| 56 | G13D | X | O | G13D | X | O |
| 57 | G12D | O | X | G12D | O | X |
| 58 | G13D | X | O | G13D | X | O |
| 59 | G13D | X | X | Wild | X | O |
| 60 | G13D | X | O | G13D | X | O |
| 61 | G12D | O | X | G12D | O | X |
| 62 | G12D | O | X | Wild | O | X |
| 63 | G13D | X | O | G13D | X | O |
| 64 | G12D | O | X | G12D | O | X |
| 65 | G12D | O | X | G12D | O | X |
| 66 | G13D | X | O | G13D | X | O |
| 67 | G13D | X | O | G13D | X | O |
| 68 | Wild | X | X | Wild | O | X |
| 69 | G13D | X | O | G13D | X | O |
| 70 | G12D | O | X | G12D | O | X |

- Highlight in blue color indicates the newly detected mutation using ISAD-KRAS assay only

- Highlight in gray color indicates the mutation detected using either ISAD-KRAS and PCR assays or ISAD-KRAS and Sequencing assays
